# Ethnic Disparities in ST-Segment Elevation Myocardial Infarction Outcomes and Processes of Care in Patients With and Without Standard Modifiable Cardiovascular Risk Factors: A Nationwide Cohort Study

**DOI:** 10.1177/00033197231182555

**Published:** 2023-06-12

**Authors:** Nicholas Weight, Saadiq Moledina, Louise Sun, Kristian Kragholm, Phillip Freeman, Carlos Diaz-Arocutipa, Mohamed Dafaalla, Martha Gulati, Mamas A. Mamas

**Affiliations:** 1 Keele Cardiovascular Research Group, Centre for Prognosis Research, Institute for Primary Care and Health Sciences, 4212 Keele University, Stoke-on-Trent, UK; 2 Division of Cardiac Anesthesiology, 27339 University of Ottawa Heart Institute, Ottawa, Ontario, Canada; 3 Department of Cardiology, 53141 Aalborg University Hospital, Aalborg, Denmark; 4 Vicerrectorado de Investigación, 33225 Universidad San Ignacio de Loyola, Lima, Peru; 5 Barbra Streisand Women’s Heart Center, 633418 Cedars-Sinai Smidt Heart Institute, Los Angeles, CA, USA

**Keywords:** STEMI, ethnicity, diabetes mellitus, hypercholesterolemia, smoking and hypertension

## Abstract

Trials suggest patients with ST-elevation myocardial infarction (STEMI) without ‘standard modifiable cardiovascular risk factors’ (SMuRFs) have poorer outcomes, but the role of ethnicity has not been investigated. We analyzed 118,177 STEMI patients using the Myocardial Ischaemia National Audit Project (MINAP) registry. Clinical characteristics and outcomes were analyzed using hierarchical logistic regression models; patients with ≥1 SMuRF (n = 88,055) were compared with ‘SMuRFless’ patients (n = 30,122), with subgroup analysis comparing outcomes of White and Ethnic minority patients. SMuRFless patients had higher incidence of major adverse cardiovascular events (MACE) (odds ratio, OR: 1.09, 95% CI 1.02–1.16) and in-hospital mortality (OR: 1.09, 95% CI 1.01–1.18) after adjusting for demographics, Killip classification, cardiac arrest, and comorbidities. When additionally adjusting for invasive coronary angiography (ICA) and revascularisation (percutaneous coronary intervention (PCI) or coronary artery bypass grafts surgery (CABG)), results for in-hospital mortality were no longer significant (OR 1.05, 95% CI .97–1.13). There were no significant differences in outcomes according to ethnicity. Ethnic minority patients were more likely to undergo revascularisation with ≥1 SMuRF (88 vs 80%, *P* < .001) or SMuRFless (87 vs 77%, *P* < .001. Ethnic minority patients were more likely undergo ICA and revascularisation regardless of SMuRF status.

## Introduction

There is growing interest in the outcomes of patients presenting with acute myocardial infarction (AMI) in the absence of ‘standard modifiable cardiovascular risk factors’ (SMuRFs), (current smoking, hypercholesterolemia, hypertension and/or diabetes mellitus).^1^ A recent analysis of the SWEDEHEART registry suggests that patients presenting with ST-segment myocardial infarction (STEMI) without SMuRFs, coined ‘SMuRFless’, have worse 30 day all-cause mortality than those with ≥1 SMuRFs.^1^ Mortality in SMuRFless patients’ post-STEMI was also found to be higher in the ‘Coronary Revascularization Demonstrating Outcome Study’ (CREDO-KYOTO) registry, suggested to be due to increased age at presentation and a higher percentage presenting in cardiogenic shock.^2^

Conversely, an analysis of non-ST segment myocardial infarction (NSTEMI) patients in the United Kingdom (UK) showed that SMuRFless patients had lower in-hospital mortality than those with ≥1 SMuRFs, although were less likely to receive guideline-directed medical therapy (GDMT), undergo invasive coronary angiography (ICA) and revascularisation.^3^ With similarities in pathophysiology, risk factors, and clinical outcomes between STEMI and NSTEMI patients, this raises the question as to why the relationship between mortality and the presence of SMuRFs varies according to whether the patients suffer STEMI or NSTEMI.^4^ One potential reason is that differences in the population demographics could be contributing to these disparate findings. Ethnic minority patients with STEMI represent a heterogeneous group with presentation at a younger age, increased burden of coronary artery disease (CAD) and higher prevalence of diabetes, hypercholesterolemia and hypertension.^5,6^

With the growing evidence suggesting increased mortality in the SMuRFless cohort, our study uses the Myocardial Ischaemia National Audit Project (MINAP) registry to investigate how STEMI outcomes vary depending on the presence of SMuRFs, and whether the outcomes and processes of care vary according to ethnicity within a nationally funded health care system.

## Methods

### Study Design

We used the MINAP, a prospective national registry of patients admitted to hospitals in the UK with acute coronary syndrome (ACS).^7^ The MINAP dataset consists of 130 variables including baseline demographics and clinical characteristics, comorbid conditions, management strategies, pharmacotherapy, place of care, in-hospital clinical outcomes and diagnoses on discharge.^8,9^ Data are submitted by hospital clinical and clerical staff and approximately 90,000 pseudonymized records annually are uploaded to the National Institute for Cardiovascular Outcomes Research (NICOR) since October 2000. Clinical outcomes beyond index hospital admission are not recorded in MINAP.

### Study Population

**Figure 1.**
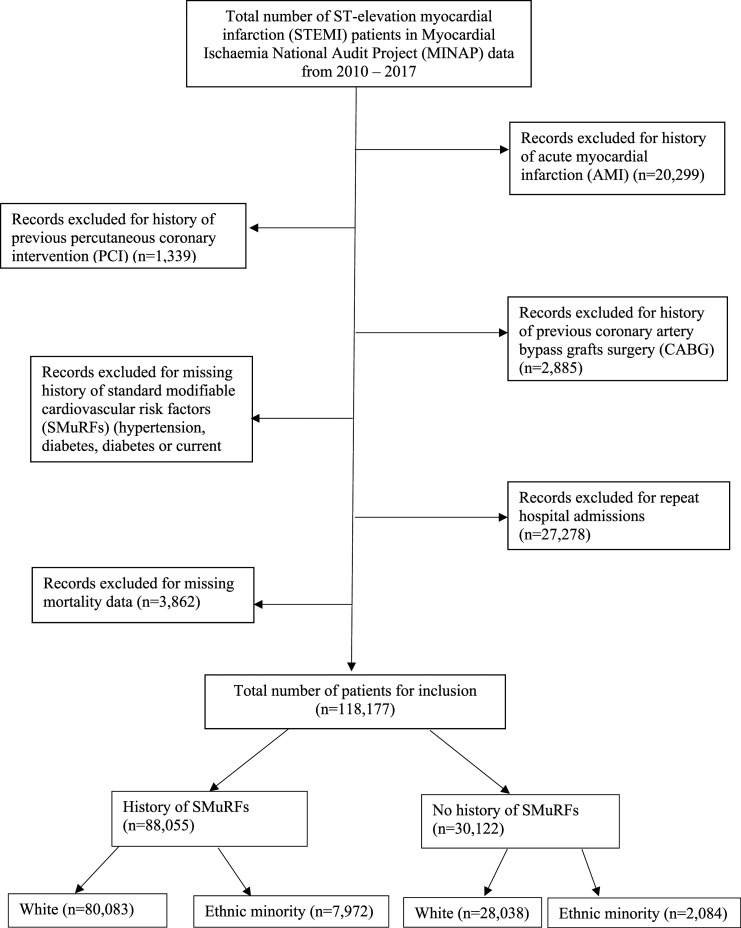
Strengthening the Reporting of Observational studies in Epidemiology (STROBE) diagram detailing exclusion criteria.

We included patients admitted with a diagnosis of STEMI in any of the 230 participating hospitals in England and Wales between 1st January 2010 to 31st March 2017, which is the most recent MINAP registry data available at present. The discharge diagnosis of STEMI was determined by local clinicians according to presenting history, clinical examination, and the results of inpatient investigations in keeping with the consensus document of the Joint European Society of Cardiology (ESC) and American College of Cardiology (ACC).^10^ Patients were excluded if they had missing data in any of the following key variables that defined the ‘SMuRFs’ group: history of hypercholesterolemia, hypertension, diabetes mellitus, and/or current smoking. To enable comparison with similar registry-based studies,^1,11^ we excluded those with a history percutaneous coronary intervention (PCI), a history of AMI, and a history of coronary artery bypass graft (CABG) surgery. Patients were also excluded if they had missing data regarding in-hospital mortality. Additionally, any individual patient’s subsequent admissions were excluded from analysis (Figure 1). This constituted a final cohort of 118,177 patients with STEMI, who were then divided into two subgroups depending on whether they had SMuRFs; group 1: with a history of at least one of the SMuRF variables and group 2 consisting of SMuRFless patients.

A history of SMuRFs was defined as the presence of ≥1 of the following variables from the MINAP dataset (as defined in the MINAP data dictionary); a history of hypertension (a patient already receiving treatment (drug, dietary, or lifestyle) for hypertension or with recorded blood pressure (BP) > 140/90 mmHg on at least two occasions prior to admission), a history of hypercholesterolemia (elevation of serum cholesterol requiring dietary or drug treatment), a history of current smoking (a current smoker is a patient regularly smoking an average of ≥1 cigarettes/day, or equivalent, any cigarettes smoked in the last month classify the patient as a current smoker), or a history of diabetes mellitus (any previous diagnosis of diabetes).

We progressed to analyze the baseline characteristics, management strategies, and outcomes of patients with and without SMuRFs stratified by ethnicity. This involved dividing our previously formed ≥1 SMuRFs and SMuRFless groups according to ethnicity. We formed two groups according to ethnicity status in the MINAP dataset: white and ethnic minority. Our Ethnic minority group comprised; Black (including Caribbean, African, Black British, any other Black background), Asian (including Indian, Pakistani, Bangladeshi, Asian British, any other Asian Background but excluding Chinese) and other non-White ethnicity (mixed group including Chinese), according to the classification of ethnicity from the MINAP data dictionary.^12^

### Outcomes

#### Primary

Primary outcomes of interest included in-hospital all-cause mortality and major adverse cardiovascular events (MACE) (composite endpoint of in-patient all-cause mortality and reinfarction).

#### Secondary

Secondary outcomes of interest included cardiac mortality (death attributable to myocardial ischemia or infarction, heart failure (HF) and cardiac arrest of unknown cause), and incidence of in-hospital major-bleeding.

### Statistical Analysis

Demographics, clinical characteristics, and crude adverse outcomes of patients by vascular bed were compared using the Pearson Chi-squared test for categorical variables. Continuous variables were compared using Student’s t-test if normally distributed and using Wilcoxon Rank Sum test if not. Normality of distribution was assessed using Shapiro–Wilks test. Continuous variables are presented as medians and interquartile ranges (IQR) and categorical variables by proportions. Multiple imputations with chained equations (MICE) were used to impute values for variables with missing data, with 10 imputations undertaken MICE is considered to be best practice when dealing with missing data, and can provide unbiased estimates even when levels of missing data are significant, and also some protection when the pattern of ‘missingness’ is not random.^13^ For each binary outcome of interest, multivariable logistic regression analysis was applied on imputed datasets to estimate the risk of adverse outcomes between groups. Estimates were combined using Rubin’s rules.^14^

Using our imputed data, adjusted odds ratios (OR) with their 95% confidence intervals (CI) were estimated using a series of hierarchical logistic regression models with patients nested within hospitals using maximum likelihood estimation. Model 1 adjusted for age, sex, year and ethnicity (for the ≥1 SMuRFs vs SMuRFless comparison only), Model 2 adjusted for these, alongside, heart rate, blood pressure, serum creatinine concentration on admission, Killip class and cardiac arrest at presentation. Model 3 adjusted for Model 2 and family history of CAD, left ventricular systolic dysfunction (LVSD), cerebrovascular accident, peripheral artery disease (PAD), asthma/chronic obstructive pulmonary disease (COPD), and Model 4 adjusted for Model 3 plus, ICA, PCI, and CABG surgery during admission. Statistical analysis was carried out using Stata 14.2 (College Station, Texas, USA). A two-tailed *P* < .05 was considered statistically significant. Tables 1 and 2.

**Table 1.** Demographic comparison between patients with ≥1 SMuRF and patients without SMuRFs stratified by ethnicity.

Variable	No SMuRFs (n = 30,122)		*p*	≥1 SMuRFs (n = 88,055)		*p*
Subgroup	White (n = 28,038)	Ethnic Minority (n = 2084)		White (n = 80,083)	Ethnic Minority (n = 7972)	
Age, years, median (IQR)	67 (5 7-78)	57 (48-68)		63 (54-74)	58 (49-68)	
Women, n (%)	7770/28,038 (28)	385/2084 (18)	<.001	24,950/80,083 (31)	1666/7972 (21)	<.001
BMI, median [IQR]	26.5 (23.8-29.4)	25.9 (23.8-28.4)		27.0 (24.1-30.4)	26.2 (23.7-29.4)	
Killip class						
No heart failure, n (%)	15,510/18,448 (84)	1251/1470 (85)	<.001	44,169/53,089 (83)	4876/5754 (85)	<.001
Basal crepitations, n (%)	1682/18,448 (9)	85/1470 (6)	<.001	5140/53, 089 (10)	380/5754 (7)	<.001
Pulmonary edema, n (%)	653/18,448 (4)	57/1470 (4)	<.001	2036/53,089 (4)	232/5754 (4)	<.001
Cardiogenic shock, n (%)	603/18,448 (3)	77/1470 (5)	<.001	1744/53,089 (3)	266/5754 (5)	<.001
GRACE–risk score						
High risk GRACE score >140, n (%)	14,075/17,219 (82)	973/1370 (71)	<.001	38,570/49,834 (77)	3880/5466 (71)	<.001
Intermediate risk GRACE score 109-140, n (%)	2737/17,219 (16)	325/1370 (24)	<.001	9530/49,834 (19)	1280/5466 (23)	<.001
Low risk GRACE score <109, n (%)	407/17,219 (2)	72/1370 (5)	<.001	1734/49,834 (3)	306/5466 (6)	<.001
Smoking						
Never smoked, n (%)	16,578/28,038 (59)	1629/2084 (78)	<.001	21,572/80,083 (27)	3355/7972 (42)	<.001
Previous smoker, n (%)	11,460/28,038 (41)	455/2084 (22)	<.001	17,257/80,083 (22)	960/7972 (12)	<.001
Current smoker, n (%)	N/A	N/A		41,254/80,083 (52)	3657/7972 (46)	<.001
Chronic renal failure, n (%)	375/27,755 (1)	19/2067 (1)	.097	2227/79,075 (3)	196/7841 (2)	.104
Diabetes, n (%)	N/A	N/A	N/A	13,942/80,083 (17)	2672/7972 (34)	<.001
CCF, n (%)	389/27,866 (1)	20/2070 (1)	.104	1222/79,387 (2)	132/7907 (2)	.372
Hypercholesterolemia, n (%)	N/A	N/A	N/A	28,533/80,083 (36)	3391/7972 (43)	<.001
History of angina, n (%)	1109/27,809 (4)	55/2068 (3)	.003	5344/79,224 (7)	484/7904 (6)	.035
Cerebrovascular disease, n (%)	915/27,841 (3)	28/2072 (1)	<.001	4114/79,310 (5)	297/7899 (4)	<.001
Peripheral artery disease, n (%)	296/27,814 (1)	8/2070 (0)	.003	2357/79,192 (3)	88/7882 (0)	<.001
Hypertension (%)	N/A	N/A	N/A	42,938/80,083 (54)	4406/7972 (55)	.005
Asthma/COPD, n (%)	3071/27,843 (11)	172/2076 (8)	<.001	9613/79,333 (12)	670/7895 (8)	<.001
Family history of CAD, n (%)	7082/24,789 (29)	527/1925 (27)	.26	25,005/71,453 (35)	2300/7251 (32)	<.001
Heart rate, bpm, median (IQR)	76 (64-89)	78 (66-89)		77 (65-90)	79 (67-90)	
Systolic blood pressure, median (IQR), mmHg	130 (112-149)	129 (110-148)		130 (113-149)	130 (110-148)	
LVSD						
Good LV function, n (%)	7707/16,736 (46)	612/1284 (48)	.105	23,659/49,154 (48)	2530/5074 (50)	.036
Moderate LVSD, n (%)	7192/16,736 (43)	555/1284 (44)		20,383/49,154 (41)	2011/5074 (40)	
Severe LVSD, n (%)	1837/16,736 (11)	117/1284 (9)		5112/49,154 (10)	533/5074 (11)	
Cardiac arrest, n (%)	3117/27,780 (11)	233/2051 (11)	.846	8667/79,071 (11)	790/7857 (10)	.014
Admission under cardiologist, n (%)	23,793/27,219 (87)	1938/2043 (95)	<.001	69,631/78,049 (89)	7434/7820 (95)	<.001
Admission to cardiology ward, n (%)	24,290/27,714 (88)	1891/2041 (93)	<.001	70,882/79,401 (89)	7333/7884 (93)	<.001

CABG; coronary artery bypass graft, LVSD; left ventricular systolic dysfunction, CAD; coronary artery disease, COPD; chronic obstructive pulmonary disease, MI; myocardial infarction, CCF; congestive cardiac failure, BMI; body mass index, GRACE; global registry of acute coronary events, IQR; interquartile range. Admission to cardiology ward is a composite of admission to coronary care unit (CCU) and general cardiology ward.

‘Good’ left ventricular systolic function is recorded in MINAP as an ejection fraction of ≥50%, “Moderate” refers to an ejection fraction of 30-49%, and “Poor” <30%.

**Table 2.** Management strategy and clinical outcome comparison between patients with ≥1 SMuRF and patients without SMuRFs stratified by ethnicity.

Variables	No SMuRFs (n = 30,122)		*P*	≥1 SMuRFs (n = 88,055)		*P*
Subgroup	White (n = 28,038)	Ethnic Minority (n = 2084)		White (n = 80,083)	Ethnic Minority (n = 7972)	
Low molecular weight heparin, n (%)	11,376/25,091 (45)	688/1828 (38)	<.001	32,112/71,780 (45)	2606/6874 (38)	<.001
Fondaparinux, n (%)	3742/24,862 (15)	263/1821 (14)	.483	9817/71,317 (14)	988/6861 (14)	.146
Warfarin, n (%)	917/24,842 (4)	48/1820 (3)	.020	2484/71,178 (3)	185/6837 (3)	.001
Unfractionated heparin, n (%)	11,145/24,814 (45)	856/1825 (47)	.099	33,553/71,204 (47)	3381/6872 (49)	.001
Glycoprotein 2b/3a inhibitor, n (%)	4780/25,553 (19)	396/1857 (21)	.005	13,828/72,931 (19)	1338/7062 (19)	.977
IV nitrate, n (%)	5429/24,823 (22)	536/1820 (29)	<.001	16,390/71,199 (23)	2010/6844 (29)	<.001
Furosemide, n (%)	4220/24,858 (17)	267/1827 (15)	.009	12,617/71,238 (18)	1096/6831 (16)	.001
Calcium channel blockers, n (%)	1696/24,795 (7)	129/1824 (7)	.705	8575/71,202 (12)	876/6843 (13)	.066
IV beta-blockers, n (%)	523/25,082 (2)	47/1827 (3)	.162	1434/71,759 (2)	144/6866 (2)	.577
MRA, n (%)	2342/24,880 (9)	169/1819 (9)	.863	6425/71,026 (9)	644/6777 (10)	.211
Thiazide diuretics, n (%)	198/24,808 (1)	10/1822 (1)	.243	1919/71,154 (3)	127/6846 (2)	<.001
Aspirin, n (%)	26,984/27,952 (97)	2022/2079 (97)	.081	76,924/79,835 (96)	7739/7955 (97)	<.001
P2Y12 inhibitor, n (%)	26,084/28,002 (93)	1970/2083 (95)	.012	75,694/79,976 (95)	7620/7962 (96)	<.001
Statins, n (%)	22,542/27,988 (81)	1751/2082 (84)	<.001	69,025/79,949 (86)	7145/7961 (90)	<.001
ACE inhibitors/ARB, n (%)	21,456/27,985 (77)	1675/2081 (80)	<.001	66,437/79,924 (83)	6821/7962 (86)	<.001
Beta-blockers, n (%)	24,023/27,902 (86)	1843/2070 (89)	<.001	69,845/79,678 (88)	7152/7935 (90)	<.001
Radionuclide study, n (%)	463/23,549 (2)	61/1530 (4)	<.001	1429/67,821 (2)	175/6092 (3)	<.001
Exercise test, n (%)	745/24,966 (3)	43/1872 (2)	.089	2180/71,633 (3)	169/7048 (2)	.002
Coronary angiogram, n (%)	24,020/27,993 (86)	1941/2083 (93)	<.001	70,808/79,961 (89)	7468/7963 (94)	<.001
Percutaneous coronary intervention, n (%)	21,506/27,965 (77)	1814/2083 (87)	<.001	64,186/79,908 (80)	7004/7967 (88)	<.001
CABG surgery, n (%)	418/20,711 (2)	29/1641 (2)	.484	1407/59,847 (2)	183/6263 (3)	.005
Revascularization (CABG surgery/PCI), n (%)	21,811/27,965 (78)	1829/2083 (88)	<.001	65,228/79,908 (82)	7119/7967 (89)	<.001
Death, n (%)	1950/28,038 (7)	100/2084 (5)	<.001	4520/80,083 (6)	364/7972 (5)	<.001
Cardiac mortality, n (%)	1761/28,038 (6)	95/2084 (5)	.002	4106/80,083 (5)	337/7972 (4)	<.001
Reinfarction, n (%)	377/27,170 (1)	24/1949 (1)	.568	1031/77,669 (1)	112/7550 (1)	.261
Major bleeding, n (%)	430/27,547 (2)	22/2061 (1)	.078	1253/78,502 (2)	115/7870 (1)	.361
MACE*, n (%)	2242/28,038 (8)	118/2084 (6)	<.001	5312/80,083 (7)	459/7972 (6)	.003

IV**;** intravenous**,** MRA**;** mineralocorticoid receptor antagonist**,** ACE**:** angiotensin-converting-enzyme**,** ARB**;** angiotensin receptor blockers**,** CABG**;** coronary artery bypass graft**,** PCI**;** percutaneous coronary intervention and MACE**;** major adverse cardiovascular events. MACE is defined as composite endpoint of in-hospital death and reinfarction.

Medication (%) is a composite of medication received during admission and prescribed at discharge.

## Results

### Baseline Characteristics for Patients With ≥1 SMuRFs *vs* SMuRFless Patients

SMuRFless patients presented older (67 [57–77] vs 63 [53–74] years) and were less likely to be female (27 vs 30%, *P* < .001) compared with those with ≥1 SMuRFs. They were less likely to have a history of cerebrovascular disease (CeVD) (3 vs 5%, *P* < .001) or PAD (1 vs 3%, *P* < .001). SMuRFless patients less frequently presented with good LV systolic function (Ejection fraction ≥50%) (46 vs 48%, *P* < .001). Patients with ≥1 SMuRFs were more frequently admitted under the care of a cardiology consultant (90 vs 88%, P < .001) and to a cardiology ward (90 vs 88%, *P* < .001) (Supplementary Table 1).

### Management Strategies for Patients With ≥1 SMuRFs *vs* SMuRFless Patients

SMuRFless patients were less likely to be prescribed P2Y12 inhibitors (93 vs 95%, *P* < .001), statins (81 vs 87%, *P* < .001), ACE inhibitors/ARBs (77 vs 83%, *P* < .001) and beta-blockers (86 vs 88%, *P* < .001) during hospitalization for STEMI. SMuRFless patients were less likely to undergo ICA (86 vs 89%, *P* < .001) or revascularisation by PCI or CABG surgery (78 vs 81%, *P* < .001). Unadjusted in-hospital mortality (7 vs 6%, *P* < .001), cardiac mortality (6 vs 5%, *P* < .001) and MACE (8 vs 7%, *P* < .001) were higher in the SmuRFless group compared with patients with ≥1 SMuRFs (Supplementary Table 2).

### Clinical Outcomes for Patients With ≥1 SMuRFs *vs* SMuRFless Patients

**Figure 2.**
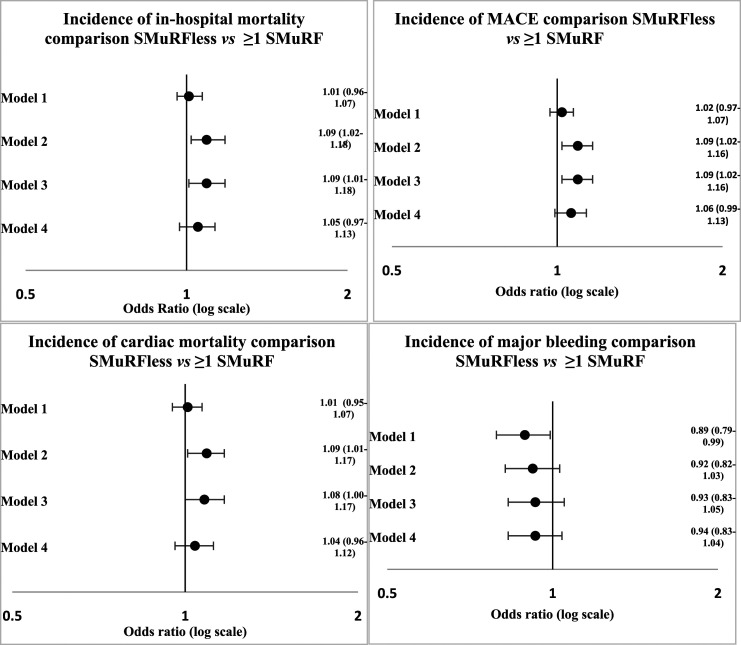
Primary Outcome comparison between SMuRFless patients and patients with SMuRFs. Figure legend: Primary Outcome comparison between SMuRFless patients and patients with SMuRFs.

Figure 2 shows our hierarchical logistic regression models applied to our primary and secondary outcomes for SMuRFless patients compared with patients with ≥1 SMuRFs. In-hospital mortality was more frequent in SMuRFless patients when adjusting for age, sex, gender, ethnicity, hemodynamics, serum creatinine, heart failure, and cardiac arrest (Model (2) (OR 1.09, 95% CI 1.02–1.18) and the aforementioned plus family history of CAD, LVSD, CeVD, PAD, asthma, and COPD (Model (3) (OR 1.09, 95% CI 1.01–1.18) but there was no statistically significant difference in mortality when additionally adjusting for ICA or revascularisation (Model (4) (OR 1.05, 95% CI .97–1.13). The incidence of MACE was higher in SMuRFless patients when applying Model 2 (OR 1.09, 95% CI 1.02–1.16) and Model 3 (OR 1.09, 95% CI 1.02–1.16), but not statistically significant when applying Model 4 (OR 1.06, 95% CI .99-1.13).

The incidence of cardiac mortality was higher in SMuRFless patients when adjusting for age, year, gender, ethnicity, hemodynamics, serum creatinine, heart failure, and cardiac arrest (Model (2) (OR 1.09, 95% CI 1.01–1.17) and further adjusting for family history of CAD, LVSD, CeVD, PAD, asthma, and COPD (Model (3) (OR 1.08, 95% CI 1.00–1.17). When adjusting for age, sex, gender, and ethnicity only, major bleeding was less likely to occur in SMuRFless patients (OR .89, 95% CI .80–.99), whereas when adjusting for any additional variables, there were no statistically significant results.

### Baseline Characteristics for Patients With ≥1 SMuRFs *vs* SMuRFless Patients Stratified by Ethnicity

Patients without SMuRFs were more likely to be White than those with ≥1 SMuRFs (93 vs 91%). Ethnic minority patients, regardless of SMuRFs status, presented younger (≥1 SMuRFs; 58 [49–68] vs 63 [54–74] years), (SMuRFless; 57 [48–68] vs 67 [57–78] years) and were less likely to be female than their White comparison groups (≥1 SMuRFs; 21 vs 31%) (SMuRFless; 18 vs 28%). Ethnic minority patients were more likely to present in cardiogenic shock (≥1 SMuRFs; 5 vs 3%) (SMuRFless; 5 vs 3%). White patients were more likely to present with a high GRACE score (≥1 SMuRFs; 77 vs 71%) (SMuRFless; 82 vs 71%). Ethnic minority patients with and without SMuRFs were more likely to be admitted to a cardiology ward (≥1 SMuRFs; 93 vs 89%) (SMuRFless; 93 vs 87%) and under the care of a consultant cardiologist (≥1 SMuRFs; 95 vs 89%), (SMuRFless; 95 vs 87%).

### Management Strategies for Patients With ≥ 1 SMuRFs *vs* SMuRFless Patients Stratified by Ethnicity

Ethnic minority patients, regardless of SMuRFs status, were more likely to receive statins (≥1 SMuRFs; 90 vs 86%), (SMuRFless; 84 vs 81%), ACE inhibitors/ARBs (≥1 SMuRFs 86 vs 83%), (SMuRFless; 80 vs 77%) and beta-blockers (≥1 SMuRFs; 90 vs 88%), (SMuRFless; 89 vs 86%) compared with White patients. Ethnic minority patients were more likely to undergo revascularisation by PCI or CABG surgery (≥1 SMuRFs; 88 vs 80%), (SMuRFless; 87 vs 77%). In unadjusted data, ethnic minority patients had a lower frequency of MACE (≥1 SMuRFs; 6 vs 7%), (SMuRFless; 6 vs 8%), in-hospital mortality (≥1 SMuRFs; 5 vs 6%), (SMuRFless; 5 vs 7%) or cardiac-mortality (≥1 SMuRFs; 4 vs 5%), (SMuRFless; 5 vs 6%).

### Clinical Outcomes Strategies for Patients With ≥ 1 SMuRFs *vs* SMuRFless Patients Stratified by Ethnicity

**Figure 3.**
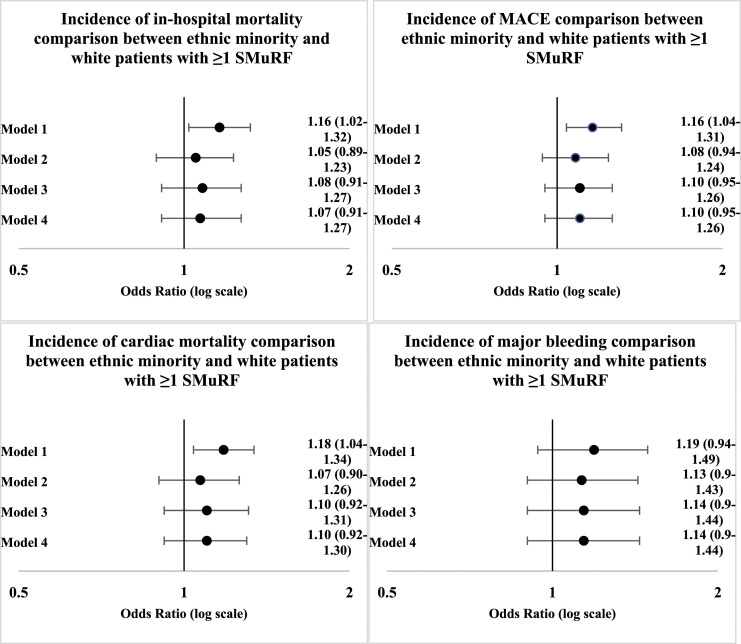
Primary Outcome comparison between ethnic minority and white patients with SMuRFs. Primary Outcome comparison between ethnic minority and white patients with SMuRFs MACE is defined as composite endpoint of in-hospital death and reinfarction.

**Figure 4.**
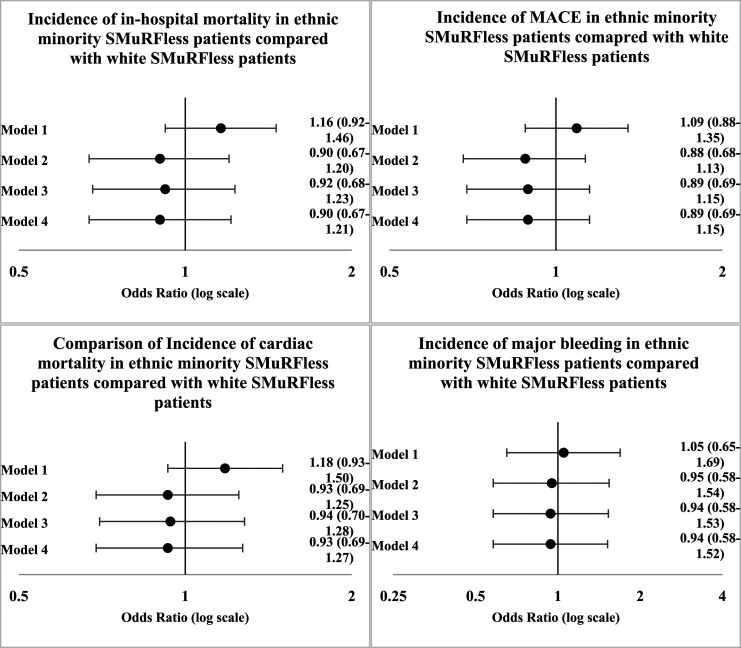
Primary Outcome comparison between ethnic minority and white SMuRFless patients. Figure legend: Primary Outcome comparison between ethnic minority and white SMuRFless patients MACE is defined as composite endpoint of in-hospital death and reinfarction.

Figure 3 shows the difference in clinical outcomes between ethnic minority patients and White patients in those that have ≥1 SMuRFs. Figure 4 shows the equivalent comparison for SMuRFless ethnic minority and White patients. When adjusting for age, sex and year only (Model 1), the likelihood of in-hospital mortality (OR 1.16, 95% CI 1.02–1.32), MACE (OR 1.16, 95% CI 1.04–1.31) and cardiac mortality (OR 1.18, 95% CI 1.04–1.34) was higher in ethnic minority patients with ≥1 SMuRFs when compared with White patients with ≥1 SMuRFs, but with adjustment for further variables, results were not statistically significant. There were no statistically significant results for comparing clinical outcomes between SMuRFless ethnic minority and White patients.

Supplementary Figure 1 illustrates clinical outcomes of White patients stratified by the presence of SMuRFs, and Supplementary Figure 2 shows this comparison for ethnic minority patients. In-hospital mortality was more likely in white SMuRFless patients compared with White patients with SMuRFs when adjusting for age, year, gender, hemodynamics, serum creatinine, heart failure and cardiac arrest (Model (2) (OR 1.10, 95% CI 1.02–1.19) and when additionally adjusting for comorbidities (OR 1.09, 95% CI 1.01–1.18), whereas the equivalent comparison for ethnic minority groups did not reach statistical significance. MACE was more likely to arise in White SMuRFless patients adjusting for age, year, gender, hemodynamics, serum creatinine, heart failure and cardiac arrest (Model (2) (OR 1.10, 95% CI 1.05–1.17), additionally adjusting for family history of CAD, LVSD, CeVD, PAD, asthma, and COPD (Model (3) (OR 1.10, 95% CI 1.02–1.15) and when further adjusting for ICA and revascularisation (Model (4) (OR 1.07, 95% CI 1.00–1.15), whereas results were not statistically significant in the ethnic minority group.

Cardiac mortality was more likely in White SMuRFless patients when adjusting for age, year, gender, hemodynamics, heart failure and cardiac arrest (Model (2) (OR 1.09, 95% CI 1.01–1.18) and when further adjusting for family history of CAD, LVSD, CeVD, PAD, asthma, and COPD (Model (3) (OR 1.08, 95% CI 1.00–1.17).

## Discussion

Our nationwide analysis of over 100,000 patients hospitalized with STEMI in the UK reveals important findings. First, SMuRFless patients were demographically different compared with patients with ≥1 SMuRFs; being older, more likely to be male and less likely to have common comorbidities such as CeVD and PAD. When stratified by ethnicity, ethnic minority patients were younger and more likely to be male, regardless of the presence of SMuRFs. Second, using a series of hierarchical models, we found that while SMuRFless patients had a slightly higher incidence of in-hospital mortality, cardiac mortality and MACE compared with patients with ≥1 SMuRFs, persisting after adjusting for baseline demographics, Killip class, cardiac arrest and common comorbidities, our study is the first to show that this increased incidence of in-hospital mortality, cardiac mortality and MACE does not persist after adjusting for differences in the provision of ICA, PCI, or CABG surgery during hospital admission. Third, we demonstrate that SMuRFless patients had significant differences in their clinical management; they were less likely to be admitted directly to cardiology wards or under a consultant cardiologist, they were less likely to be treated with GDMT with P2Y12 inhibitors, statins, ACE inhibitors/ARBs and were less likely to undergo ICA or PCI during admission. Fourth, in the first analysis of the role of ethnicity in outcomes of SMuRFless and patients with ≥1 SMuRFs in the UK, we found that ethnic minorities were not disadvantaged in processes of care compared with White patients. Finally, there was no statistically significant relationship between ethnicity and our primary outcomes in SMuRFless patients.

Figtree et al demonstrated the increased 30-day mortality of SMuRFless patients in their analysis of the SWEDEHEART registry.^1^ Their group had previously demonstrated the increasing proportion of patients with STEMI that were presenting in the absence of SMuRFs, suggesting that the profile of patients suffering STEMI was changing from what we had previously considered as being at particularly high risk, and that these SMuRFless patients also had a higher in-hospital mortality than those with ≥1 SMuRFs.^11,15^ In an analysis of the CREDO-KYOTO PCI/CABG registry, it was shown that crude all-cause mortality was higher in SMuRFless patients but noted that after adjusting for common confounders such as age, hemodynamic status and medical therapy, there was no statistically significant difference between SMuRF groups with STEMI and NSTEMI but did note increased mortality in their cardiogenic shock subgroup.^2^ This showed similarity with our results, with our small, demonstrated increases in in-hospital mortality, MACE, and cardiac mortality in SMuRFless patients progressively declining after adjusting for more variables with our hierarchical models, losing statistical significance after adjusting for ICA and revascularisation strategy. This suggests that there are a range of factors contributing to the increased observed mortality in SMuRFless patients, but that a potential area for improvement in the outcomes of these patients would be improving the processes of care, particularly with an invasive strategy and the use of GDMT in SMuRFless patients.

The observed increases in mortality in SMuRFless patients following AMI is in marked contrast to the prevailing understanding of cardiovascular risk factors informed by the INTERHEART study, which suggests that a significant proportion of AMI is attributable to nine modifiable risk factors, including all the SMuRF risk factors.^16^ One consideration is whether the SMuRFless patients are truly SMuRFless, or whether they have just avoided detection prior to admission. The YOUNG-MI registry investigated the prevalence of risk factors in infarction in patients suffering AMI under the age of 50 in multiple US centers, and found that 20% of patients had hypercholesterolemia diagnosed in their index admission with AMI, with diabetes first diagnosed in 22% and hypertension in 9% of patients.^17^ This suggests that a significant proportion of our SMuRFless group may be subsequently diagnosed with one of the standard modifiable cardiovascular risk factors during their index admission, but due to the nature of the MINAP registry data collection, we are unable to adjust for this.

Our results are consistent with those of Figtree et al with regards to discharge pharmacotherapy, highlighting how SMuRFless patients are less likely to be discharged on statins, ACE inhibitors, beta-blockers, and DAPT.^1^ They suggested this discrepancy in discharge pharmacotherapy to be a possible mechanism of the increased 30-day mortality in SMuRFless patients, and this could contribute to the in-hospital mortality of patients with longer hospital stays pre-discharge in our study. The benefits of early pharmacotherapy with ACE inhibition, beta-blockers, and statins are well demonstrated to improve mortality in patients suffering AMI, and it is clear that this is an area we should be aiming to improve in the management of SMuRFless STEMI patients.^18-20^

Our analysis is one of the first to assess whether ethnicity influences the differences in STEMI outcomes according to SMuRF status. In our hierarchical models, we show a small increase in adjusted in-hospital mortality, MACE, and cardiac mortality in White SMuRFLess patients compared with white patients with ≥1 SMuRFs, but that this does not persist after adjusting for ICA and revascularisation strategy. The equivalent comparison for ethnic minority patients was showed no statistically significant relationship. In fact, patients from an ethnic minority background were found to have a reduced likelihood of in-hospital death, cardiac mortality, or MACE in our unadjusted data when compared with white patients, were more likely to undergo revascularisation by PCI or CABG and more likely to be treated with P2Y12 inhibitors, beta-blockers, ACE inhibitors/ARBs, and statin therapy. This is consistent with previous work from our group when evaluating the impact of ethnicity on the outcomes of NSTEMI within the UK, where ethnic minority patients were more likely to be prescribed GDMT, undergo ICA and revascularisation.^7^ We suspect that higher prevalence of comorbidities such as hypertension and diabetes mellitus found in the UK ethnic minority group is leading to a greater perception of risk from CAD, leading to more aggressive treatment in this NSTEMI cohort, but this does not fully explain such high rates of GDMT and revascularisation in our SMuRFless ethnic minority cohort.^21^

The results from the UK contrast with those from the US, with a recent analysis of the National Inpatient Sample (NIS) where in 159,399 STEMI patients with cardiogenic shock, Black and Hispanic patients had higher in-hospital mortality when compared with White patients, and demonstrated that Black patients were less likely to undergo revascularisation compared with white patients.^5^ Consistently, studies from the US, show lower rates of GDMT prescription, ICA and revascularisation in ethnic minority groups, which may relate to differences in healthcare funding and equity of access across the two healthcare systems.^22^ Contemporary studies have now consistently demonstrated an elevated mortality in SMuRFless patients, including large registries from across the world, including the UK, Sweden, Japan, and China, across a range of ethnicities, it is therefore unlikely that the underlying cause for this elevation in mortality is ethnicity-driven.^23^ It is interesting to note the increased mortality in white SMuRFless patients compared with White patients with SMuRFs in our study, but that this is not the case for the same comparison in ethnic minority patients. A likely reason for this is the greater proportion of females in the White cohort compared with ethnic minority patients in our study, which is in keeping with previous studies of the NSTEMI cohort.^7^ This is in keeping with the work of Figtree et al suggesting that the increased mortality in SMuRFless patients is more pronounced in female patients.^1^ A potential reason for this is the higher proportion of female patients that present with rarer causes of STEMI, such as spontaneous coronary artery dissection (SCAD) or takotsubo cardiomyopathy, where there is a higher early mortality, and less effective evidence-based therapy.^24-26^ This is likely to be an important contributor to the increased mortality in the white SMuRFless group compared with those with SMuRFs. Although we excluded patients with takotsubo cardiomyopathy from our analysis, there is always the risk of misclassification in our registry data, which could influence our results. Further studies should be undertaken to identify the reasons for this elevated mortality risk, so that these high-risk patients can be better targeted with early diagnosis of modifiable cardiovascular risk factors, primary prevention and GDMT.

There are important limitations to observational studies like the present one. The MINAP data collection shares the weakness of other national registries, including self-reporting of adverse events with no external validation. Although the MINAP dataset includes important clinical and demographic variables of interest, there are limitations to data collected, such as the lack of frailty score, severity of CAD, socioeconomic factors, access to healthcare, rationale for specific medications or a full list of comorbidities. Our current definition of hypercholesterolemia based upon serum cholesterol levels may not fully capture all patients at risk, with LDL-C levels having been demonstrated to be independently associated with the extent and severity of systemic atherosclerosis, even when at levels that are currently considered at normal.^27^ The comorbidities in MINAP are typically recorded on admission, meaning that we are unable to include patients diagnosed with a modifiable risk factor during admission, which has meant that our SMuRFless cohort is larger than comparable studies. Patients that were recorded as previous smokers were included to remain consistent with comparable studies, although we acknowledge that these patients may not be truly risk-factor free. The MINAP registry does not record data regarding outcomes following discharge, collecting only in-hospital outcomes, so we are unfortunately unable to replicate the analysis of comparable studies using registries such as SWEDEHEART.^1^ We are unable to differentiate between pre-hospital pharmacotherapy, and medications started as an inpatient from our registry data which is particularly relevant given a potential cause for increased mortality in SMuRFless patients is pre-admission pharmacotherapy.^28,29^ Due to the demographics of the UK, our sample sizes of the ethnic groups that comprised our ethnic minority group; Black, Asian, mixed and other, were too small with which to carry out a meaningful analysis. We acknowledge that due to the heterogeneity of the ethnic minority group, further differences in the risk factor profiles and outcomes of ACS may not have been fully gleaned.^30^ Population-based cohort studies from Scotland have demonstrated how the ethnicities within our ethnic minority group have different healthcare outcomes, with Pakistani patients identified as high risk of AMI and mortality, whereas Chinese patients typically have lower rates of AMI and better outcomes.^31,32^ We acknowledge that the ethnic minority group is markedly smaller than the white group, which means our study may not be sufficiently powered to detect statistically significant differences in outcomes between groups.

## Conclusion

In our nationwide study of 118,177 patients with STEMI in the UK, we demonstrate that SMuRFless patients have a higher incidence of MACE, in-hospital mortality, and cardiac-mortality than those with ≥1 SMuRFs, which persists after adjusting for baseline demographics, hemodynamic status, Killip classification, cardiac arrest, and common comorbidities, but does not persist after adjusting for ICA or revascularisation. Ethnic minority patients were more likely to be younger, male and receive GDMT, undergo ICA and revascularisation regardless of SMuRF status. After adjusting for hemodynamic status, serum creatinine, Killip classification and cardiac arrest, there was no significant difference in our primary or secondary outcomes between White and ethnic minority patients. Further studies should be undertaken to identify these high-risk SMuRFless patients that do not have, or have undiagnosed conventional cardiovascular risk factors, and efforts made to improve awareness of the importance of GDMT for these patients.

## Supplemental Material


Supplemental Material - Supplemental Material for Ethnic Disparities in ST-Segment Elevation Myocardial Infarction Outcomes and Processes of Care in Patients With and Without Standard Modifiable Cardiovascular Risk Factors: A Nationwide Cohort Study
Supplemental Material for Supplemental Material for Ethnic Disparities in ST-Segment Elevation Myocardial Infarction Outcomes and Processes of Care in Patients With and Without Standard Modifiable Cardiovascular Risk Factors: A Nationwide Cohort Study by Nicholas Weight, Saadiq Moledina, Louise Sun, Kristian Kragholm, Phillip Freeman, Carlos Diaz-Arocutipa, Mohamed Dafaalla, Martha Gulati, and Mamas A. Mamas in Angiology

## Appendix

### Abbreviations list

AMI: Acute myocardial infarction
CABG: Coronary artery bypass grafts
CAD: Coronary artery disease
CeVD: Cerebrovascular disease
COPD: Chronic obstructive pulmonary disease
CI: Confidence interval
GDMT: Guideline directed medical therapy
ICA: Invasive coronary angiography
LVSD: Left ventricular systolic function
MACE: Major adverse cardiovascular events
MINAP: Myocardial Ischaemia National Audit Project
OR: Odds ratio
PAD: Peripheral artery disease
PCI: Percutaneous coronary intervention
SMuRFs: Standard modifiable cardiovascular risk factors
STEMI: ST-elevation myocardial infarction

## References

[1] Figtree GA Vernon ST Hadziosmanovic N , et al. Mortality in STEMI patients without standard modifiable risk factors: A sex-disaggregated analysis of SWEDEHEART registry data. Lancet. 2021;397(10279):1085-1094. 33711294 10.1016/S0140-6736(21)00272-5

[2] Yamamoto K Natsuaki M Morimoto T , et al. Coronary artery disease without standard cardiovascular risk factors. Am J Cardiol. 2022;164:34-43. 34852931 10.1016/j.amjcard.2021.10.032

[3] Moledina SM Rashid M Nolan J , et al. Addressing disparities of care in non-ST-segment elevation myocardial infarction patients without standard modifiable risk factors: Insights from a nationwide cohort study. Eur J Prev Cardiol. 2022;29:1084-1092. 34897399 10.1093/eurjpc/zwab200

[4] Montalescot G Dallongeville J Van Belle E , et al. STEMI and NSTEMI: Are they so different? 1 year outcomes in acute myocardial infarction as defined by the ESC/ACC definition (the OPERA registry). Eur Heart J. 2007;28(12):1409-1417. 17412730 10.1093/eurheartj/ehm031

[5] Ya'qoub L Lemor A Dabbagh M , et al. Racial, ethnic, and sex disparities in patients with STEMI and cardiogenic shock. JACC Cardiovasc Interv. 2021;14(6):653-660. 33736772 10.1016/j.jcin.2021.01.003

[6] Kim EJ Kressin NR Paasche-Orlow MK , et al. Racial/ethnic disparities among Asian Americans in inpatient acute myocardial infarction mortality in the United States. BMC Health Serv Res. 2018;18(1):370. 29769083 10.1186/s12913-018-3180-0 PMC5956856

[7] Moledina SM Shoaib A Weston C , et al. Ethnic disparities in care and outcomes of non-ST-segment elevation myocardial infarction: A nationwide cohort study. Eur Heart J Qual Care Clin Outcomes. 2022;8:518-528. 33892502 10.1093/ehjqcco/qcab030

[8] Wilkinson C Weston C Timmis A Quinn T Keys A Gale CP . The myocardial ischaemia national audit project (MINAP). Eur Heart J Qual Care Clin Outcomes. 2020;6(1):19-22. 31511861 10.1093/ehjqcco/qcz052

[9] Rashid M Curzen N Kinnaird T , et al. Baseline risk, timing of invasive strategy and guideline compliance in NSTEMI: Nationwide analysis from MINAP. Int J Cardiol. 2020;301:7-13. 31810815 10.1016/j.ijcard.2019.11.146

[10] Alpert JS Thygesen K Antman E Bassand JP . Myocardial infarction redefined-a consensus document of The Joint European Society of cardiology/American college of cardiology committee for the redefinition of myocardial infarction. J Am Coll Cardiol. 2000;36(3):959-969. 10987628 10.1016/s0735-1097(00)00804-4

[11] Vernon ST Coffey S D'Souza M , et al. ST-segment-elevation myocardial infarction (STEMI) patients without standard modifiable cardiovascular risk factors-how common are they, and what are their outcomes? J Am Heart Assoc. 2019;8(21):e013296. 31672080 10.1161/JAHA.119.013296 PMC6898813

[12] Moledina SM Kontopantelis E Wijeysundera HC , et al. Ethnicity-dependent performance of the global registry of acute coronary events risk score for prediction of non-ST-segment elevation myocardial infarction in-hospital mortality: nationwide cohort study. Eur Heart J. 2022;43:2289-2299. 35202472 10.1093/eurheartj/ehac052

[13] Kontopantelis E White IR Sperrin M Buchan I . Outcome-sensitive multiple imputation: A simulation study. BMC Med Res Methodol. 2017;17(1):2. 28068910 10.1186/s12874-016-0281-5 PMC5220613

[14] Rubin DB . Multiple imputation after 18+ years. J Am Stat Assoc. 1996;91(434):473-489.

[15] Vernon ST Coffey S Bhindi R , et al. Increasing proportion of ST elevation myocardial infarction patients with coronary atherosclerosis poorly explained by standard modifiable risk factors. Eur J Prev Cardiol. 2017;24(17):1824-1830. 28703626 10.1177/2047487317720287

[16] Yusuf S Hawken S Ounpuu S , et al. Effect of potentially modifiable risk factors associated with myocardial infarction in 52 countries (the INTERHEART study): Case-control study. Lancet. 2004;364(9438):937-952. 15364185 10.1016/S0140-6736(04)17018-9

[17] Singh A Collins BL Gupta A , et al. Cardiovascular risk and statin eligibility of young adults after an MI: Partners YOUNG-MI registry. J Am Coll Cardiol. 2018;71(3):292-302. 29141201 10.1016/j.jacc.2017.11.007 PMC5831171

[18] ISIS-4: A randomised factorial trial assessing early oral captopril, oral mononitrate, and intravenous magnesium sulphate in 58,050 patients with suspected acute myocardial infarction. ISIS-4 (fourth international study of infarct survival) collaborative group. Lancet. 1995;345(8951):669-685. 7661937

[19] Fonarow GC Wright RS Spencer FA , et al. Effect of statin use within the first 24 hours of admission for acute myocardial infarction on early morbidity and mortality. Am J Cardiol. 2005;96(5):611-616. 16125480 10.1016/j.amjcard.2005.04.029

[20] Bugiardini R Cenko E Ricci B , et al. Comparison of early versus delayed oral beta blockers in acute coronary syndromes and effect on outcomes. Am J Cardiol. 2016;117(5):760-767. 26778165 10.1016/j.amjcard.2015.11.059

[21] Graham G . Racial and ethnic differences in acute coronary syndrome and myocardial infarction within the united states: from demographics to outcomes. Clin Cardiol. 2016;39(5):299-306. 27028198 10.1002/clc.22524 PMC6490862

[22] Wadhera RK Bhatt DL Kind AJH , et al. Association of outpatient practice-level socioeconomic disadvantage with quality of care and outcomes among older adults with coronary artery disease: Implications for value-based payment. Circ Cardiovasc Qual Outcomes. 2020;13(4):e005977. 32228065 10.1161/CIRCOUTCOMES.119.005977 PMC7259485

[23] Kong G Chew NWS Ng CH , et al. Prognostic outcomes in acute myocardial infarction patients without standard modifiable risk factors: A multiethnic study of 8,680 Asian patients. Front Cardiovasc Med. 2022;9:869168. 35425823 10.3389/fcvm.2022.869168 PMC9001931

[24] Garcia-Guimaraes M Bastante T Antuna P , et al. Spontaneous coronary artery dissection: Mechanisms, diagnosis and management. Eur Cardiol. 2020;15:1-8. 10.15420/ecr.2019.01 PMC7113739 32256714

[25] Macaya F Salinas P Gonzalo N Fernandez-Ortiz A Macaya C Escaned J . Spontaneous coronary artery dissection: Contemporary aspects of diagnosis and patient management. Open Heart. 2018;5(2):e000884. 30487978 10.1136/openhrt-2018-000884 PMC6241978

[26] Templin C Ghadri JR Diekmann J , et al. Clinical features and outcomes of takotsubo (stress) cardiomyopathy. N Engl J Med. 2015;373(10):929-938. 26332547 10.1056/NEJMoa1406761

[27] Fernandez-Friera L Fuster V Lopez-Melgar B , et al. Normal LDL-cholesterol levels are associated with subclinical atherosclerosis in the absence of risk factors. J Am Coll Cardiol. 2017;70(24):2979-2991. 29241485 10.1016/j.jacc.2017.10.024

[28] Volpe M Patrono C . The increased mortality of STEMI patients without risk factors supports the need for evidence-based pharmacotherapy irrespective of perceived low risk. Eur Heart J. 2021;42(24):2329-2330. 34153988 10.1093/eurheartj/ehab268

[29] Wang JY Goodman SG Saltzman I , et al. Cardiovascular risk factors and in-hospital mortality in acute coronary syndromes: Insights from the canadian global registry of acute coronary events. Can J Cardiol. 2015;31(12):1455-1461. 26143140 10.1016/j.cjca.2015.04.007

[30] Lu HT Nordin RB . Ethnic differences in the occurrence of acute coronary syndrome: Results of the Malaysian National cardiovascular disease (NCVD) database registry (March 2006-February 2010). BMC Cardiovasc Disord. 2013;13:97. 24195639 10.1186/1471-2261-13-97 PMC4229312

[31] Katikireddi SV Cezard G Bhopal RS , et al. Assessment of health care, hospital admissions, and mortality by ethnicity: Population-based cohort study of health-system performance in Scotland. Lancet Public Health. 2018;3(5):e226-e236. 29685729 10.1016/S2468-2667(18)30068-9 PMC5937910

[32] Bansal N Fischbacher CM Bhopal RS , et al. Myocardial infarction incidence and survival by ethnic group: Scottish health and ethnicity linkage retrospective cohort study. BMJ Open. 2013;3(9):e003415. 10.1136/bmjopen-2013-003415 PMC3773657 24038009

