# Supplementary material for: Ethnic Disparities in ST-Segment Elevation Myocardial Infarction Outcomes and Processes of Care in Patients With and Without Standard Modifiable Cardiovascular Risk Factors: A Nationwide Cohort Study
Source: Angiology. 2023 Jun 12;75(8):742–53. doi: 10.1177/00033197231182555 (PMC11311903; doi:10.1177/00033197231182555)
Supplement: Supplemental Material - Supplemental Material for Ethnic Disparities in ST-Segment Elevation Myocardial Infarction Outcomes and Processes of Care in Patients With and Without Standard Modifiable Cardiovascular Risk Factors: A Nationwide Cohort Study [file sj-pdf-1-ang-10.1177_00033197231182555.pdf]

Supplementary Table 1: Demographic comparison between patients with >=1 SMuRF and patients without SMuRFs.

**Variable**	**No SMuRFs (n=30,122)**	**>=1 SMuRFs (n=88,055)**	**p**
Age, years, median (IQR)	67 (57-77)	63 (53-74)	<0.001
Women, n (%)	8,155/30,122 (27)	26,616/88,055 (30)	<0.001
BMI, median [IQR]	26.5 (23.8-29.4)	27.0 (24.1-30.4)	<0.001
Ethnicity- White, n (%)	28,038/30,122 (93)	80,083/88,055 (91)	<0.001
Ethnicity- Ethnic minority, n (%)	2,084/30,122 (7)	7,972/88,055 (9)	<0.001
**Killip Class**			
No heart failure, n (%)	16,761/19,918 (84)	49,045/58,843 (83)	0.035
Basal crepitations, n (%)	1,767/19,918 (9)	5,520/58,843 (9)	
Pulmonary edema, n (%)	710/19,918 (4)	2,268/58,843 (4)	
Cardiogenic shock, n (%)	680/19,918 (3)	2,010/58,843 (3)	
**GRACE – risk score**			
High risk GRACE score >140, n (%)	15,048/18,589 (81)	42,450/55,300 (77)	<0.001
Intermediate risk GRACE score 109-140, n (%)	3,062/18,589 (16)	10,810/55,300 (20)	
Low risk GRACE score <109, n (%)	479/18,589 (3)	2,040/55,300 (4)	
**Smoking**			
Never smoked, n (%)	18,207/30,122 (60)	24,927/88,055 (28)	<0.001
Previous smoker, n (%)	11,915/30,122 (40)	18,217/88,055 (21)	

Current smoker, n (%)	N/A	44,911/88,055 (51)	
Chronic renal failure, n (%)	394/29,822 (1)	2,423/86,916 (3)	<0.001
Diabetes, n (%)	(0)	16,614/88,055 (19)	<0.001
CCF, n (%)	409/29,936 (1)	1,354/87,294 (2)	0.023
Hypercholesterolemia, n (%)	(0)	31,924/88,055 (36)	<0.001
History of angina, n (%)	1,164/29,877 (4)	5,828/87,128 (7)	<0.001
Cerebrovascular disease, n (%)	943/29,913 (3)	4,411/87,209 (5)	<0.001
Peripheral artery disease, n (%)	304/29,884 (1)	2,445/87,074 (3)	<0.001
Hypertension (%)	(0)	47,344/88,055 (54)	<0.001
Asthma / COPD, n (%)	3,243/29,919 (11)	10,283/87,228 (12)	<0.001
Family history of CAD (%)	7,609/26,714 (28)	27,305/78,704 (35)	<0.001
Heart rate, bpm, median (IQR)	76 (64-89)	77 (65-90)	<0.001
Systolic blood pressure, median (IQR), mmHg	130 (114-149)	130 (113-150)	0.7855
**LVSD**			
Good LV function, n (%)	8,319/18,020 (46)	26,189/54,228 (48)	<0.001
Moderate LVSD, n (%)	7,747/18,020 (43)	22,394/54,228 (41)	
Severe LVSD, n (%)	1,954/18,020 (11)	5,645/54,228 (10)	
Cardiac arrest, n (%)	3,350/29,831 (11)	9,457/86,928 (11)	0.094
Admission under cardiologist, n (%)	25,731/29,262 (88)	77,065/85,869 (90)	<0.001
Admission to cardiology ward, n (%)	26,181/29,755 (88)	78,215/87,285 (90)	<0.001

CABG; coronary artery bypass graft, LVSD; left ventricular systolic dysfunction, CAD; coronary artery disease, COPD; chronic obstructive pulmonary disease, MI; myocardial infarction, CCF; congestive cardiac failure, BMI; body mass index, GRACE; global registry of acute coronary

events, IQR; interquartile range. Admission to cardiology ward is a composite of admission to coronary care unit (CCU) and general cardiology ward.

‘Good’ left ventricular systolic function is recorded in MINAP as an ejection fraction of ≥50%, ‘Moderate’ refers to an ejection fraction of 30-49%, and ‘Poor’ <30%.

Supplementary Table 2: Management strategy and clinical outcome comparison between patients with ≥1 SMuRF and patients without SMuRFs.

**Variables**	**No SMuRFs (n=30,122)**	**≥1 SMuRFs (n=88,055)**	**p**
Low molecular weight heparin, n (%)	12,064/26,919 (45)	34,718/78,654 (44)	0.054
Fondaparinux, n (%)	4,005/26,683 (15)	10,805/78,178 (14)	<0.001
Warfarin, n (%)	965/26,662 (4)	2,669/78,015 (3)	0.127
Unfractionated heparin, n (%)	12,001/26,639 (45)	36,934/78,076 (47)	<0.001
Glycoprotein 2b/3a inhibitor, n (%)	5,176/27,410 (19)	15,166/79,993 (19)	0.783
IV Nitrate, n (%)	5,965/26,643 (22)	18,400/78,043 (24)	<0.001
Furosemide, n (%)	4,487/26,685 (17)	13,713/78,069 (18)	0.005

Calcium channel blockers, n (%)	1,825/26,619 (7)	9,451/78,045 (12)	<0.001
IV beta-blockers, n (%)	570/26,909 (2)	1,578/78,625 (2)	0.265
MRA, n (%)	2,511/26,699 (9)	7,069/77,803 (9)	0.119
Thiazide diuretics, n (%)	208/26,630 (1)	2,046/78,000 (3)	<0.001
Aspirin, n (%)	29,006/30,031 (97)	84,663/87,790 (96)	0.227
P2Y12 inhibitor (%)	28,054/30,085 (93)	83,314/87,938 (95)	<0.001
Statins, n (%)	24,293/30,070 (81)	76,170/87,910 (87)	<0.001
ACE inhibitors/ARB, n (%)	23.131/30,066 (77)	73,258/87,886 (83)	<0.001
Beta-Blockers, n (%)	25,866/29,972 (86)	76,997/87,613 (88)	<0.001
Radionuclide Study, n (%)	524/25,079 (2)	1,604/73,913 (2)	0.446
Exercise test, n (%)	788/26,838 (3)	2,349/78,681 (3)	0.681
Coronary angiogram (%)	25,961/30,076 (86)	78,276/87,924 (89)	<0.001

Percutaneous coronary intervention, n (%)	23,320/30,048 (78)	71,190/87,875 (81)	<0.001
CABG surgery (%)	447/22,352 (2)	1,590/66,110 (2)	<0.001
Revascularization (CABG surgery/PCI), n (%)	23,640/30,048 (79)	72,347/87,875 (82)	<0.001
Death, n (%)	2,050/30,122 (7)	4,884/88,055 (6)	<0.001
Cardiac mortality, n (%)	1,856/30,122 (6)	4,443/88,055 (5)	<0.001
Reinfarction, n (%)	401/29,119 (1)	1,143/85,219 (1)	0.647
Major bleeding, n (%)	452/29,608 (2)	1,368/86,372 (2)	0.494
MACE*, n (%)	2,360/30,122 (8)	5,771/88,055 (7)	<0.001

IV; intravenous, MRA; mineralocorticoid receptor antagonist, ACE: angiotensin-converting-enzyme, ARB; angiotensin receptor blockers, CABG; coronary artery bypass graft, PCI; percutaneous coronary intervention and MACE; major adverse cardiovascular events. MACE is defined as composite endpoint of in-hospital death and reinfarction.
Medication (%) is a composite of medication received during admission and prescribed at discharge.

Additional Tables

Supplementary Table 3: Demographic comparison between patients with ≥1 SMuRF and patients without SMuRFs stratified by ethnicity.

Variable	No SMuRFs (n=30,122)				≥1 SMuRFs (n=88,055)			
**Subgroup**	**White (n=28,038)**	**Black (n=156)**	**Asian (n=1,224)**	**Mixed (n=704)**	**White (n=80,083)**	**Black (n=774)**	**Asian (n=4,821)**	**Mixed (n=2,377)**
Age, years, median (IQR)	67 (57-78)	55 (47-67)	56 (46-65)	61 (51-71)	63 (54-74)	57 (48-69)	58 (48-68)	58 (49-68)
Women, n (%)	7,770/28,038 (28)	31/156 (20)	207/1,224 (17)	147/704 (21)	24,950/80,083 (31)	199/774 (26)	959/4,821 (20)	508/2,377 (21)
BMI, median [IQR]	26.5 (23.8-29.4)	25.1 (22.7-27.8)	25.9 (23.1-28.1)	26.3 (24.9-29.3)	27.0 (24.1-30.4)	26.6 (24.1-31.3)	26.1 (23.7-29.3)	26.5 (23.8-29.4)
No heart failure, n (%)	15,510/18,448 (84)	81/97 (84)	732/870 (84)	438/503 (87)	44,169/53,089 (83)	501/568 (88)	2,912/3,483 (84)	1,463/1,703 (86)
Basal crepitations, n (%)	1,682/18,448 (9)	10/97 (10)	57/870 (7)	18/503 (4)	5,140/53, 089 (10)	33/568 (6)	270/3,483 (8)	77/1,703 (5)
Pulmonary oedema, n (%)	653/18,448 (4)	2/97 (2)	33/870 (4)	22/503 (4)	2,036/53,089 (4)	15/568 (3)	146/3,483 (4)	71/1,703 (4)
Cardiogenic shock, n (%)	603/18,448 (3)	4/97 (4)	48/870 (6)	25/503 (5)	1,744/53,089 (3)	19/568 (3)	155/3,483 (4)	92/1,703 (5)
High risk GRACE score >140, n (%)	14,075/17,219 (82)	58/89 (65)	545/811 (67)	370/470 (79)	38,570/49,834 (77)	353/537 (66)	2,314/3,315 (70)	1,213/1,614 (75)
Intermediate risk GRACE score 109-140,	2,737/17,219 (16)	25/89 (28)	215/811 (27)	85/470 (18)	9,530/49,834 (19)	152/537 (28)	794/3,315 (24)	334/1,614 (21)

n (%)								
Low risk GRACE score, n <109 (%)	407/17,219 (2)	6/89 (7)	51/811 (6)	15/470 (3)	1,734/49,834 (3)	32/537 (6)	207/3,315 (6)	67/1,614 (4)
Never smoked, n (%)	16,578/28,038 (59)	123/156 (79)	1,018/1,224 (83)	488/704 (69)	21,572/80,083 (27)	300/774 (39)	2,332/4,821 (48)	733/2,377 (31)
Previous smoker, n (%)	11,460/28,038 (41)	33/156 (21)	206/1,224 (17)	216/704 (31)	17,257/80,083 (22)	103/774 (13)	539/4,821 (11)	318/2,377 (13)
Current smoker, n (%)	N/A	N/A	N/A	N/A	41,254/80,083 (52)	371/774 (48)	1,960/4,821 (41)	1,326/2,377 (56)
Chronic renal failure, n (%)	375/27,755 (1)	4/154 (3)	8/1,212 (1)	7/701 (1)	2,227/79,075 (3)	22/764 (3)	132/4,717 (3)	42/2,360 (2)
Diabetes, n (%)	N/A	N/A	N/A	N/A	13,942/80,083 (17)	238/774 (31)	1,892/4,821 (39)	542/2,377 (23)
CCF, n (%)	389/27,866 (1)	2/154 (1)	8/1,214 (1)	10/702 (1)	1,222/79,387 (2)	12/767 (2)	70/4,777 (1)	50/2,363 (2)
Hypercholesterolemia, n (%)	N/A	N/A	N/A	N/A	28,533/80,083 (36)	300/774 (39)	2,240/4,821 (46)	851/2,377 (36)
History of angina, n (%)	1,109/27,809 (4)	5/152 (3)	35/1,215 (3)	15/701 (2)	5,344/79,224 (7)	44/767 (6)	328/4,771 (7)	112/2,366 (5)
Cerebrovascular disease, n (%)	915/27,841 (3)	0/155 (0)	14/1,217 (1)	14/700 (2)	4,114/79,310 (5)	50/765 (7)	184/4,766 (4)	73/2,368 (3)
Peripheral vascular disease, n (%)	296/27,814 (1)	1/154 (1)	3/1,215 (0)	4/701 (1)	2,357/79,192 (3)	20/763 (3)	49/4,759 (1)	19/2,360 (1)
Hypertension, n (%)	N/A	N/A	N/A	N/A	42,938/80,083 (54)	474/774 (61)	2,762/4,821 (57)	1,170/2,377 (49)
Asthma / COPD, n (%)	3,071/27,843 (11)	7/155 (5)	99/1,218 (8)	66/703 (9)	9,613/79,333 (12)	63/765 (8)	411/4,767 (9)	196/2,363 (8)

Family history of CAD, n (%)	7,082/24,789 (29)	24/141 (17)	362/1,152 (31)	141/632 (22)	25,005/71,453 (35)	140/713 (20)	1,574/4,434 (35)	586/2,104 (28)
Heart rate, bpm, median (IQR)	76 (64-89)	77 (70-88)	78 (67-90)	77 (65-87)	77 (65-90)	78 (66-90)	80 (68-92)	77 (65-90)
Systolic blood pressure, median (IQR)	130 (112-149)	127 (111-152)	130 (110-147)	131 (115-150)	130 (113-149)	132 (116-151)	130 (112-148)	130 (111-150)
Good LV function, n (%)	7,707/16,736 (46)	46/94 (49)	358/725 (49)	208/465 (45)	23,659/49,154 (48)	261/508 (51)	1,544/2,960 (52)	725/1,606 (45)
Moderate LVSD, n (%)	7,192/16,736 (43)	35/94 (37)	305/725 (42)	215/465 (46)	20,383/49,154 (41)	189/508 (37)	1,117/2,960 (38)	705/1,606 (44)
Severe LVSD, n (%)	1,837/16,736 (11)	13/94 (14)	62/725 (9)	42/465 (10)	5,112/49,154 (10)	58/508 (11)	299/2,960 (10)	176/1,606 (11)
Cardiac arrest, n (%)	3,117/27,780 (11)	27/151 (18)	126/1,206 (10)	80/694 (12)	8,667/79,071 (11)	67/757 (9)	457/4,742 (10)	266/2,358 (11)
Admission under cardiologist, n (%)	23,793/27,219 (87%)	143/154 (93)	1,132/1,200 (94)	663/689 (96)	69,631/78,049 (89)	723/755 (96)	4,417/4,715 (94)	2,294/2,350 (98)
Admission to cardiology ward, n (%)	24,290/27,714 (88)	128/153 (84)	1,119/1,195 (94)	644/693 (93)	70,882/79,401 (89)	706/762 (93)	4,381/4,757 (92)	2,246/2,365 (95)

CABG; coronary artery bypass graft, LVSD; left ventricular systolic dysfunction, CAD; coronary artery disease, COPD; chronic obstructive pulmonary disease, MI; myocardial infarction, CCF; congestive cardiac failure, BMI; body mass index, GRACE; global registry of acute coronary events, IQR; interquartile range. Admission to cardiology ward is a composite of admission to coronary care unit (CCU) and general cardiology ward.

Supplementary Table 4: Management strategy and clinical outcome comparison between patients with ≥1 SMuRF and patients without SMuRFs stratified by ethnicity.

Variables	No SMuRFs (n=30,122)				≥1 SMuRFs (n=88,055)			
**Subgroup**	**White (n=28,038)**	**Black (n=156)**	**Asian (n=1,224)**	**Mixed (n=704)**	**White (n=80,083)**	**Black (n=774)**	**Asian (n=4,821)**	**Mixed (n=2,377)**
Low molecular weight heparin, n (%)	11,376/25,091 (45)	58/134 (43)	367/1,041 (35)	263/653 (40)	32,112/71,780 (45)	270/693 (39)	1,527/3,984 (38)	809/2,197 (37)
Fondaparinux, n (%)	3,742/24,862 (15)	25/134 (19)	115/1,035 (11)	123/652 (19)	9,817/71,317 (14)	88/693 (13)	540/3,978 (14)	360/2,190 (16)
Warfarin, n (%)	917/24,842 (4)	7/131 (5)	23/1,037 (2)	18/652 (3)	2,484/71,178 (3)	31/680 (5)	97/3,969 (2)	57/2,188 (3)
Unfractionated heparin, n (%)	11,145/24,814 (45)	56/132 (42)	480/1,040 (46)	320/653 (49)	33,553/71,204 (47)	295/693 (43)	1,981/3,988 (50)	1,105/2,191 (50)
Glycoprotein 2b/3a inhibitor, n (%)	4,780/25,553 (19)	29/136 (21)	233/1,063 (22)	134/658 (20)	13,828/72,931 (19)	127/714 (18)	801/4,139 (19)	410/2,209 (19)
IV Nitrate, n (%)	5,429/24,823 (22)	35/133 (26)	282/1,037 (27)	219/650 (34)	16,390/71,199 (23)	174/680 (26)	1,119/3,975 (28)	717/2,189 (33)
Furosemide, n (%)	4,220/24,858 (17)	15/133 (11)	157/1,042 (15)	95/652 (15)	12,617/71,238 (18)	99/679 (15)	674/3,966 (17)	323/2,186 (15)
Calcium channel blockers, n (%)	1,696/24,795 (7)	12/132 (9)	87/1,040 (8)	30/652 (5)	8,575/71,202 (12)	114/679 (17)	571/3,973 (14)	191/2,191 (9)
IV beta-blockers, n (%)	523/25,082 (2)	3/135 (1)	32/1,041 (3)	12/651 (2)	1,434/71,759 (2)	15/687 (2)	112/3,990 (3)	17/2,189 (1)
MRA, n (%)	2,342/24,880 (9)	16/134 (12)	81/1,037 (8)	72/648 (11)	6,425/71,026 (9)	66/671 (10)	314/3,924 (8)	264/2,182 (12)
Thiazide diuretics, n (%)	198/24,808 (1)	0/133 (0)	7/1,038 (7)	3/651 (0)	1,919/71,154 (3)	23/681 (3)	79/3,975 (2)	25/2,190 (1)
Aspirin, n (%)	26,984/27,952 (97)	149/155 (96)	1,184/1,211 (98)	689/703 (98)	76,924/79,835 (96)	742/771 (96)	4,685/4,809 (97)	2,312/2,375 (97)

P2Y12 inhibitor, n (%)	26,084/28,002 (93)	141/156 (90)	1,160/1,233 (94)	669/704 (95)	75,694/79,976 (95)	726/773 (94)	4,627/4,813 (96)	2,267/2,376 (95)
Statins, n (%)	22,542/27,988 (81)	121/156 (78)	1,075/1,223 (88)	555/703 (79)	69,025/79,949 (86)	716/772 (93)	4,405/4,817 (91)	2,024/2,372 (85)
ACE inhibitors/ARB, n (%)	21,456/27,985 (77)	113/156 (72)	1,030/1,223 (84)	532/702 (76)	66,437/79,924 (83)	680/772 (88)	4,208/4,818 (87)	1,933/2,372 (81)
Beta-Blockers, n (%)	24,023/27,902 (86)	132/152 (87)	1,076/1,217 (88)	635/701 (91)	69,845/79,678 (88)	686/767 (89)	4,309/4,799 (90)	2,157/2,369 (91)
Radionuclide Study, n (%)	463/23,549 (2)	5/118 (4)	32/938 (3)	24/474 (5)	1,429/67,821 (2)	13/625 (2)	105/3,794 (3)	57/1,673 (3)
Exercise test (%)	745/24,966 (3)	2/139 (1)	29/1,100 (3)	12/633 (2)	2,180/71,633 (3)	16/700 (2)	112/4,271 (3)	41/2,077 (2)
Coronary angiogram, n (%)	24,020/27,993 (86)	139/156 (89)	1,137/1,223 (93)	665/704 (94)	70,808/79,961 (89)	734/774 (95)	4,465/4,812 (93)	2,269/2,377 (95)
Percutaneous coronary intervention, n (%)	21,506/27,965 (77)	126/156 (81)	1,053/1,223 (86)	635/704 (90)	64,186/79,908 (80)	680/774 (88)	4,146/4,816 (86)	2,178/2,377 (92)
CABG surgery, n (%)	418/20,711 (2)	2/129 (2)	16/968 (2)	11/544 (2)	1,407/59,847 (2)	11/638 (2)	125/3,782 (3)	47/1,843 (3)
Revascularization (CABG surgery/PCI), n (%)	21,811/27,965 (78)	126/156 (81)	1,064/1,223 (87)	639/704 (91)	65,228/79,908 (82)	686/774 (89)	4,234/4,816 (88)	2,199/2,377 (93)
Death, n (%)	1,950/28,038 (7)	14/156 (9)	58/1,224 (5)	28/704 (4)	4,520/80,083 (6)	32/774 (4)	226/4,821 (5)	106/2,377 (4)
Cardiac mortality, n (%)	1,761/28,038 (6)	13/156 (8)	55/1,224 (4)	27/704 (4)	4,106/80,083 (5)	26/774 (3)	212/4,821 (4)	99/2,377 (4)
Reinfarction, n (%)	377/27,170 (1)	2/145 (1)	16/1,158 (1)	6/646 (1)	1,031/77,669 (1)	11/743 (1)	74/4,595 (2)	27/2,212 (1)
Major bleeding,	430/27,547	2/155 (1)	12/1,211	8/695	1,253/78,502	16/766 (2)	76/4,757	23/2,347 (1)

n (%)	(2)		(1)	(1)	(2)		(2)	
MACE*, n (%)	2,242/28,038 (8)	15/156 (10)	70/1,224 (6)	33/704 (5)	5,312/80,083 (7)	40/774 (5)	289/4,821 (6)	130/2,377 (5)

IV**;** intravenous**,** MRA**;** mineralocorticoid receptor antagonist**,** ACE**:** angiotensin-converting-enzyme**,** ARB**;** angiotensin receptor blockers**,** CABG**;** coronary artery bypass graft**,** PCI**;** percutaneous coronary intervention and MACE**;** major adverse cardiovascular events. MACE is defined as composite endpoint of in-hospital death and reinfarction.

Medication (%) is a composite of medication received during admission and prescribed at discharge.

Supplementary Table 5: Multivariate analysis comparing odds of primary and secondary outcomes comparing SMuRFless patients with patients with ≥1 SMuRFs.

**Outcome variables**	**SMuRFless patients (n=30,122) compared with patients with ≥1 SMuRFs (n=88,055) as baseline**	
	**OR (95% CIs)**	**p**
**Primary Outcomes**		
**MACE (In-hospital)**	1.06 (1.00-1.13)	0.057
**Mortality (In-hospital)**	1.06 (0.99-1.14)	0.110
**Secondary Outcomes**		
**Cardiac mortality (In-hospital)**	1.05 (0.97-1.13)	0.239
**Major bleeding (In-hospital)**	0.94 (0.84-1.04)	0.235

Adjusted for: age, sex, year, ethnicity heart rate, blood pressure, serum creatinine concentration on admission, Killip class, cardiac arrest, family history of coronary artery disease (CAD), Left ventricular systolic dysfunction (LVSD), cerebrovascular accident, peripheral vascular disease,

asthma/chronic obstructive pulmonary disease (COPD), invasive coronary angiography (ICA), percutaneous coronary intervention (PCI) and coronary artery bypass graft surgery (CABG) surgery during admission.

*MACE is defined as composite endpoint of in-hospital death and reinfarction.

*SMuRFs refers to ‘standard modifiable cardiovascular risk factors’ (including hypertension, diabetes, hypercholesterolaemia and current smoking)

Supplement Figures

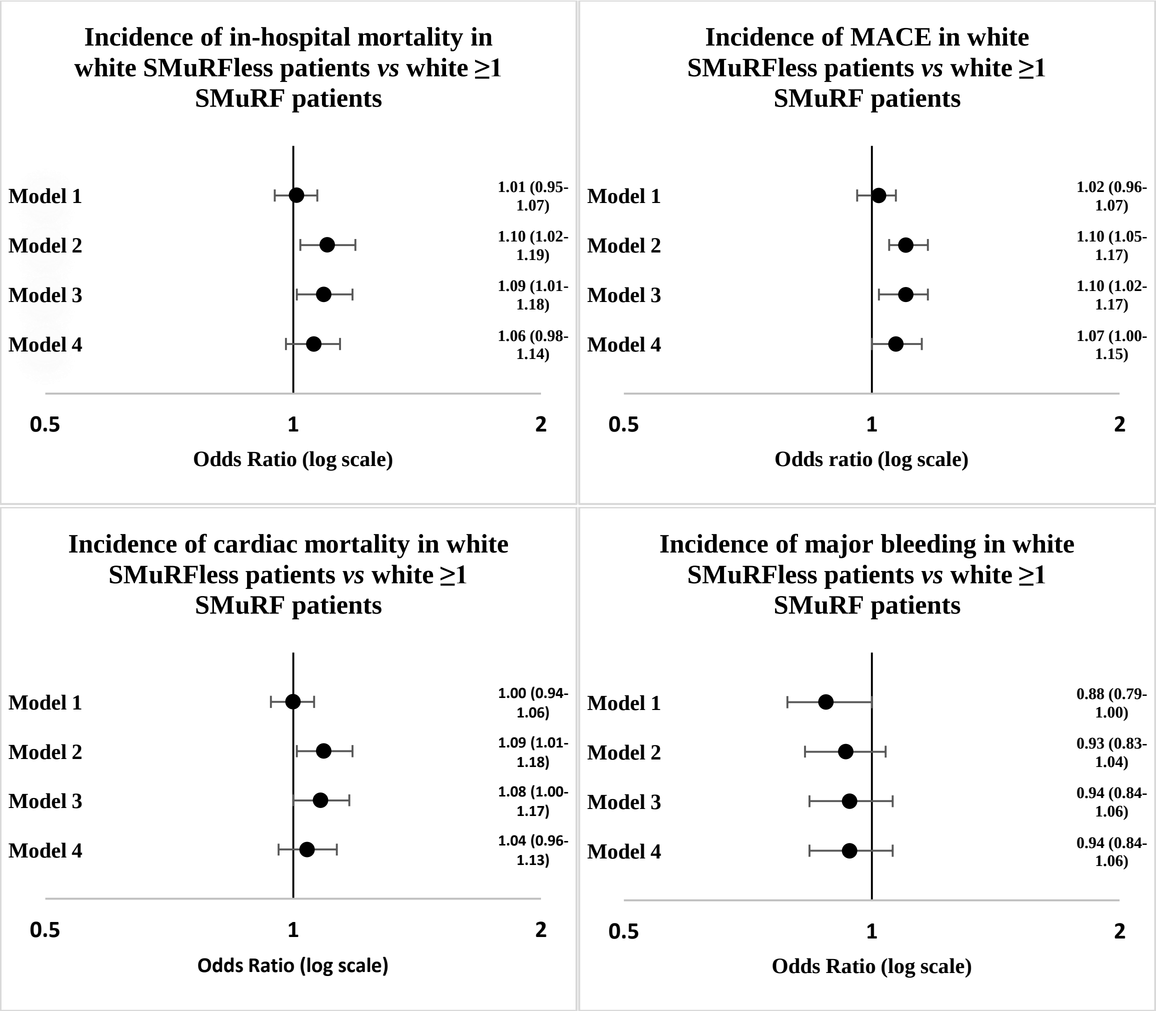

Supplementary Figure 1: Primary outcome comparison between white SMuRFless patients and white patients with SMuRFs

Using imputed data, we created a series of hierarchical logistic regression models with patients nested within hospitals using maximum likelihood estimation, adjusting for;

*Model 1: age, sex, ethnicity and year.

**Model 2: Model 1 + heart rate, blood pressure, serum creatinine concentration on admission, Killip class, cardiac arrest.

***Model 3: Model 2+ family history of coronary artery disease (CAD), left ventricular systolic dysfunction (LVSD), cerebrovascular accident, peripheral vascular disease, asthma/chronic obstructive pulmonary disease (COPD).

**** Model 4: Model 3 + invasive coronary angiography (ICA), percutaneous coronary intervention (PCI) and coronary artery bypass grafts (CABG) surgery during admission.

*MACE is "Major adverse cardiovascular events" and is defined as composite endpoint of in-hospital death and reinfarction.

*SMuRFs refers to 'standard modifiable cardiovascular risk factors' (including hypertension, diabetes, hypercholesterolaemia and current smoking)

Figure legend: Primary outcome comparison between white SMuRFless patients and white patients with SMuRFs

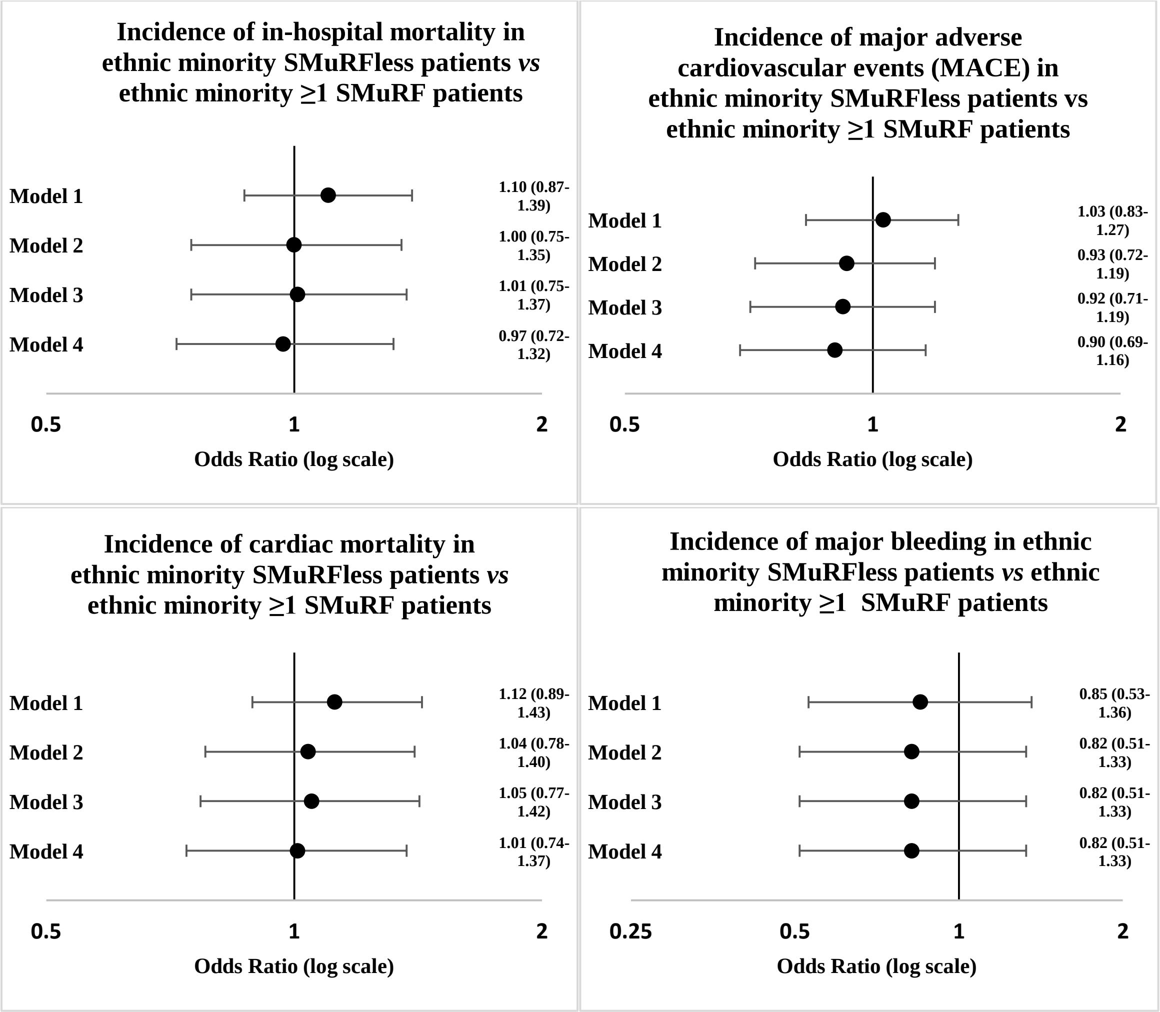

Supplementary Figure 2: Primary outcome comparison between ethnic minority SMuRFless patients and ethnic minority patients with SMuRFs

Using imputed data, we created a series of hierarchical logistic regression models with patients nested within hospitals using maximum likelihood estimation, adjusting for;

*Model 1: age, sex, and year.

**Model 2: Model 1 + heart rate, blood pressure, serum creatinine concentration on admission, Killip class, cardiac arrest.

***Model 3: Model 2+ family history of coronary artery disease (CAD), left ventricular systolic dysfunction (LVSD), cerebrovascular accident, peripheral vascular disease, asthma/chronic obstructive pulmonary disease (COPD).

**** Model 4: Model 3 + invasive coronary angiography (ICA), percutaneous coronary intervention (PCI) and coronary artery bypass grafts (CABG) surgery during admission.

*MACE is "Major adverse cardiovascular events" and is defined as composite endpoint of in-hospital death and reinfarction.

*SMuRFs refers to 'standard modifiable cardiovascular risk factors' (including hypertension, diabetes, hypercholesterolaemia and current smoking)

Figure legend: Primary outcome comparison between ethnic minority SMuRFless patients and ethnic minority patients with SMuRFs
